# Coronary microvascular disease in heart failure with preserved ejection fraction

**DOI:** 10.14814/phy2.70521

**Published:** 2025-08-20

**Authors:** Payel Sen, Lili Wang, Laura d'Ambrosio, Susanne Bierschenk, Jules Hamers, Irem Ornek, Theresa Sittig, Hengliang Zhang, Junqing Zhang, Daphne Merkus

**Affiliations:** ^1^ Institute for Surgical Research Walter Brendel Center of Experimental Medicine, University Clinic Munich Munich Germany; ^2^ Interfaculty Center for Endocrine and Cardiovascular Disease Network Modelling and Clinical Transfer (ICONLMU) University Clinic Munich Munich Germany; ^3^ German Center for Cardiovascular Research (DZHK), Munich Heart Alliance (MHA), Partner Site Munich Munich Germany; ^4^ Department of Cardiology ErasmusMC Rotterdam The Netherlands

**Keywords:** coronary microvascular disease, endothelial dysfunction, heart failure with preserved ejection fraction, mitochondria

## Abstract

The prevalence of heart failure with preserved ejection fraction (HFpEF) is increasing because of an aging population and an unhealthy, sedentary lifestyle, predisposing to diabetes, obesity, dyslipidemia, hypertension, and chronic kidney disease. A substantial proportion of patients with HFpEF exhibits coronary microvascular disease (CMD), and the combination of CMD with left ventricular diastolic dysfunction is associated with worse outcomes. Distinct patient clusters within HFpEF populations are based on clinical and biomarker profiles, each with unique prognoses and comorbidity patterns. Most patients with HFpEF present with multiple comorbidities, adding to the disease's complexity. Since risk factors directly affect the coronary microvasculature—and considering the bidirectional paracrine signaling between cardiomyocytes and the microvasculature—there is significant pathophysiological overlap between HFpEF and CMD. Therefore, in this review, we aim to summarize epidemiological evidence for the overlap in patients with CMD and HFpEF, identify shared biomarkers pathophysiological pathways underlying the co‐occurrence of CMD and HFpEF, and discuss how cardiometabolic interventions may simultaneously address both CMD and HFpEF. Established and emerging treatments for HFpEF and CMD target these shared mechanisms. A deeper understanding of these interrelated pathways may pave the way for novel therapeutic strategies to alleviate the burden of HFpEF and refine patient management.

## 1 INTRODUCTION

The prevalence of heart failure with preserved ejection fraction (HFpEF) is increasing worldwide, and lifetime risk for HFpEF development currently exceeds the risk of development of heart failure with reduced ejection fraction (HFrEF) (Abdin et al., 2024). The increasing prevalence is due to an aging population and a global pandemic of an unhealthy sedentary lifestyle, predisposing to diabetes, obesity, dyslipidemia, hypertension, chronic kidney disease (CKD), and chronic obstructive lung disease (COPD) (Borlaug et al., 2023). Comorbidities are rarely present alone; most HFpEF patients show multiple comorbidities; 25% of HFpEF patients present with one to three comorbidities, 54% with four to six comorbidities, and 21% with more than six comorbidities (Tomasoni et al., 2024). These comorbidities create a systemic pro‐inflammatory environment that leads to endothelial dysfunction and oxidative stress (Paulus & Tschope, 2013). The consequent loss of nitric oxide (NO) in the coronary microcirculation reduces cGMP in the cardiomyocytes, leading to impaired protein kinase G (PKG) activation, which promotes hypertrophy and hypophosphorylation of titin. The resulting stiffening of the cardiomyocytes, in combination with interstitial fibrosis, impairs diastolic relaxation characteristic for the development of HFpEF (Paulus & Tschope, 2013). Many studies have identified a large overlap in patients with coronary microvascular disease (CMD) and patients with HFpEF (Aldiwani et al., 2022; Arkowski et al., 2024; Chen et al., 2024; Dryer et al., 2018; Erhardsson et al., 2023; Loffler et al., 2019; Markley et al., 2023; Mohammed et al., 2015; Paolisso et al., 2024; Shah et al., 2018; Srivaratharajah et al., 2016; Taqueti et al., 2018; Wolsk et al., 2024). Indeed, risk factors for HFpEF can also directly impact the coronary microvasculature and contribute to the development of CMD by altering microvascular function and/or structure (Padro et al., 2020). Furthermore, the close anatomical relationship between cardiomyocytes and the microvasculature enables dynamic paracrine signaling in both directions. Cardiomyocytes can influence coronary blood flow, while signals from the vasculature can modulate myocardial contraction and relaxation, underscoring the interdependence of vascular and myocardial health.

HFpEF is not a single disease entity, but a complex syndrome, defined by an ejection fraction over 50%, characterized by increased left ventricular filling pressures caused by structural or functional cardiac abnormalities (Borlaug et al., 2023). Machine learning analyses of clinical risk profiles (Murray et al., 2022) as well as biomarkers in blood (Woolley et al., 2021) have revealed several patient clusters encompassing (1) patients with a high prevalence of diabetes and CKD, (2) elderly patients with age‐related comorbidities, (3) younger obese patients, and (4) patients with COPD. Between these groups, patients from group 1 show the highest activation of inflammatory pathways, group 3 has an immunometabolic profile, while group 4 shows a more ischemic phenotype, with the highest NT‐proBNP and troponin levels. Groups 1 and 4 have the worst prognosis, with a high risk of hospitalization and heart failure‐related mortality (Woolley et al., 2021). Group 3 shows that adipose tissue is not an innocent bystander but comprises metabolically active tissue with significant paracrine and inflammatory effects. Both visceral and epicardial adipose tissue (VAT, EAT) can induce systemic and local inflammation contributing to oxidative stress, microvascular injury, cardiomyocyte hypertrophy, and myocardial fibrosis (Schiattarella et al., 2022).

In this review, we aim to (1) summarize epidemiological evidence for the overlap in patients with CMD and HFpEF, (2) identify pathophysiological pathways underlying the co‐occurrence of CMD and HFpEF, and (3) describe how and why cardiometabolic interventions may ameliorate both CMD and HFpEF.

## 2 CORONARY MICROVASCULAR DISEASE AND HFPEF: EPIDEMIOLOGY

### 2.1 DEFINING CORONARY MICROVASCULAR DISEASE

The heart and the coronary microvasculature are closely interconnected. The coronary microcirculation, defined as coronary blood vessels smaller than 500 μm in diameter, comprises a densely distributed network of branching blood vessels that supplies the cardiac muscle with oxygen and nutrients. Since the myocardium utilizes already 75%–80% of the oxygen supplied by the coronary vasculature under resting conditions, changes in oxygen demand of the myocardium, for example, during exercise, must be adequately met by changes in oxygen supply to prevent myocardial ischemia. Progressive dilation of the coronary microvasculature can increase coronary blood flow up to fivefold in the healthy heart. A complex interplay of myriad vasodilators and vasoconstrictors mediates this tight regulation of myocardial oxygen supply by modulating the tone of the coronary small arteries and arterioles (Duncker et al., 2015, 2022). In response to shifts in myocardial workload, cardiomyocytes release a variety of signaling molecules and metabolic by‐products which play a key role in vasodilation. Endothelial cells are critical regulators of vascular tone, producing potent vasodilators such as nitric oxide (NO) and prostacyclin, and reduced the production of the potent vasoconstrictor endothelin‐1 in response to shear stress and other hemodynamic stimuli. Erythrocytes, acting as oxygen sensors, contribute to vascular regulation by releasing ATP and NO under hypoxic conditions. Platelets, upon activation, release a dual spectrum of vasoactive substances, including vasodilators like serotonin and vasoconstrictors such as thromboxane A_2_. The autonomic nervous system also exerts significant influence through its nerve terminals, which release neurohumoral agents like acetylcholine and norepinephrine (Duncker et al., 2015). The release and activity of these vasoactive agents are regulated through multiple overlapping mechanisms:

- Mechanical influences, such as shear stress and transmural pressure;
- Metabolic signals, including oxygen tension, CO_2_ levels, pH, and local metabolites;
- Neurohumoral control, mediated by circulating hormones and autonomic innervation.

Exposure to risk factors results in alterations in both macro‐ and microvascular function and/or structure. CMD can occur both in the presence and absence of a stenosis in the epicardial coronary arteries and contributes significantly to the myocardial ischemic burden (Padro et al., 2020; Verma et al., 2025). Functionally, altered production of vasodilators and vasoconstrictors as well as impaired smooth muscle responses to these dilators and constrictors are thought to play a role in CMD. Structurally, capillary rarefaction reduces capillary density, and (peri)vascular fibrosis may limit vasodilation (Del Buono et al., 2021; Padro et al., 2020). Moreover, diastolic dysfunction and increased heart rate increase extravascular compression and reduce diastolic perfusion time of particularly the subendocardium (Padro et al., 2020).

Clinically, CMD is diagnosed by measuring the vasodilator capacity of the coronary microvasculature by measuring coronary blood flow (CBF) at baseline and in response to endothelium‐dependent (e.g. acetylcholine) and/or endothelium‐independent (e.g. adenosine, dipyridamole) vasodilators (Verma et al., 2025). CBF can be measured invasively using a flow wire that measures flow velocity in combination with diameter measurement using angiography, or using thermodilution, using injections of saline of known temperature in combination with a temperature sensor placed distally in the coronary arteries calculating mean transit time (Tm) as an index of flow (Candreva et al., 2021). Coronary flow reserve (CFR) is calculated as the ratio of hyperemic CBF to baseline CBF or alternatively reported as the ratio of baseline Tm to hyperemic Tm. Distal intracoronary pressure (Pd) is measured simultaneously with CBF or Tm, for calculation of the coronary microvascular resistance allowing calculation of the index of microvascular resistance (IMR, product of Pd and hyperemic Tm), offering a quantitative measure of resistance within the coronary microcirculation that is not affected by epicardial disease. Myocardial perfusion can also be measured non‐invasively, most commonly using positron emission tomography (PET) or cardiomagnetic resonance imaging (CMR), allowing quantification of myocardial perfusion per gram tissue (Di Carli, 2025; Petretta et al., 2008) at baseline and after inducing vasodilation with adenosine, resulting in quantification of myocardial perfusion reserve (MPR).

Clinically, CMD is defined as signs and symptoms of ischemia in combination with a CFR below 2–2.5 in the absence of a flow limiting epicardial stenosis (luminal obstruction <50%), and/or a hyperemic IMR > 25. A CFR below 2.5, in combination with a hyperemic IMR below 25, suggests a high basal CBF and is indicative for functional CMD. A CFR below 2.5 with a hyperemic IMR above 25 denotes structural CMD (Pompei et al., 2024) and a CFR above 2.5 with an IMR above 25 is indicative for compensated structural CMD (Verma et al., 2025) although this term is less commonly used. The endotype of CMD can be further refined by assessing CFR and IMR in response to the endothelium‐dependent vasodilator acetylcholine. In the presence of endothelial dysfunction, the smooth muscle constrictor response to acetylcholine may override the endothelium‐dependent vasodilation, and focal vasospasms in response to acetylcholine can be observed both in the macro‐ and microvasculature. However, the cut‐off value for impaired endothelium‐dependent vasodilation varies between studies, ranging from <50% increase (Sara et al., 2019) to no increase at all (Nardone et al., 2022).

### 2.2 PREVALENCE OF CMD ENDOTYPES IN PATIENTS WITH ANGINA AND NO OBSTRUCTIVE CORONARY ARTERY DISEASE

In the ILIAS registry, a multinational cohort study of patients with angina and no obstructive coronary artery disease (ANOCA), compensated structural CMD was observed in 10% and structural CMD in 20% (Boerhout et al., 2022). In a smaller study, patients with low CFR/low IMR were younger and presented with fewer traditional coronary risk factors, while patients with low CFR/high IMR showed a higher septal thickness, a higher prevalence of congestive heart failure, and a trend toward a worse exercise capacity (Nardone et al., 2022). These data suggest that patients with low CFR/low IMR, as compared to patients with low CFR/ high IMR, have a lower cardiovascular risk profile and reduced risk of future adverse cardiac events. In a comparison between men and women with ANOCA, women were shown to have a lower resting IMR and CFR, while hyperemic IMR was not different (Kobayashi et al., 2015). However, such differences were not observed in the ILIAS registry (Boerhout et al., 2022).

Endothelium‐dependent CMD is more prevalent than endothelium‐independent CMD among patients with ANOCA (Woudstra et al., 2024). A large cohort study showed that 52% of ANOCA patients exhibited endothelium‐dependent CMD, 25% showed endothelium‐independent CMD, and 15% of patients had both endothelium‐dependent and independent CMD (Kanaji et al., 2024). The majority of patients (82%) with low CFR/low IMR in response to adenosine were women as a result of higher resting CBF and similar hyperemic CBF (Nardone et al., 2022).

### 2.3 PREVALENCE OF CMD IN HFPEF COHORTS

Patients with HFpEF showed a high prevalence of CMD (73%) in an exploratory study, with 37% of patients having structural CMD as evidenced by a low CFR and a high IMR, 27% with a high CFR and high IMR, and 10% with a low CFR and a low IMR, indicative of functional CMD (Dryer et al., 2018). In support of a high prevalence of structural CMD, postmortem histology of HFpEF myocardium showed reduced capillary density, which correlated with increased myocardial fibrosis (Mohammed et al., 2015). Moreover, the presence of CMD was associated with higher troponin levels (Chandramouli et al., 2022), indicative of myocardial ischemia, as well as with worse cardiac function, (Arnold et al., 2022) reduced cardiac functional reserve, (Ahmad et al., 2021) and increased mortality (Arnold et al., 2022). In another study measuring endothelium‐dependent and endothelium‐independent CMD in HFpEF patients, CMD was observed in 72% of the patients. Only 10% of the patients exhibited both endothelium‐dependent and endothelium‐independent CMD. Endothelium‐dependent CMD was associated with diabetes and lower levels of HDL cholesterol (Yang et al., 2020). These results were confirmed in several studies, and two recent meta‐analyses estimated a CMD prevalence of 71% in patients with HFpEF (ranging from 40% to 86% in individual studies), with 62% (range 40%–76%) of patients exhibiting endothelium‐independent CMD, and 50% (range 24%–86%) of patients had endothelium‐dependent CMD (D'Amario et al., 2024; Lin et al., 2024). Patients with CMD also exhibited higher pulmonary vascular resistance and right ventricular systolic pressures, suggesting a more generalized microvascular problem (Dryer et al., 2018).

### 2.4 CMD AS A RISK FACTOR FOR HFPEF DEVELOPMENT

In the WISE‐CVD cohort, CFR was inversely related to LV‐EDP (Jones et al., 2020). Furthermore, in patients with unexplained cardiac exertion symptoms, subsequently diagnosed with CMD and/or HFpEF, endothelium‐independent CMD correlated with a higher pulmonary arterial wedge pressure (PAWP), suggestive of impaired LV function at rest and during exercise, whereas endothelium‐dependent CMD only predicted a higher PAWP during exercise. Moreover, CFR and the increase in CBF during exercise correlated with peak exercise capacity (Ahmad et al., 2021). These data show that CMD is associated with impaired diastolic function and functional reserve. However, although a wealth of evidence supports the co‐existence of CMD and HFpEF, this evidence is particularly derived from HFpEF cohorts, whereas follow‐up for development of HFpEF in CMD cohorts is limited. In the DIAST‐CMD study, which included patients with CMD and/or diastolic dysfunction, patients with both CMD and diastolic dysfunction showed the highest prevalence of heart failure and cardiovascular events during follow‐up (52%) (Hong et al., 2023). In this cohort, also diastolic dysfunction and CMD alone showed a high cardiovascular event rate (41% and 33%, respectively) versus 17% in the group without CMD and diastolic dysfunction (Hong et al., 2023). Similarly, in a cohort of patients undergoing CFR measurement for evaluation of ANOCA, CFR inversely correlated with diastolic dysfunction as measured with ultrasound (Taqueti et al., 2018) and MRI (Samuel et al., 2021). Other predictors for diastolic dysfunction at baseline were impaired kidney function and atrial fibrillation. Moreover, circulating troponin levels were slightly higher in patients with CFR < 2 as compared to CFR > 2. During a follow‐up period of 4 years, patients with low CFR and diastolic dysfunction were more likely to develop HFpEF (hazard ratio of 2.4, annualized rate of HFpEF of 15% in patients with low CFR and diastolic dysfunction) (Taqueti et al., 2018). These data are in accordance with the WISE study, showing that low CFR was a risk factor for HFpEF development albeit at a low prevalence of 3% (Leong et al., 2021). In a smaller cohort, in which both CFR and IMR were measured, diastolic dysfunction was more prominent in patients with low CFR, but not in patients with high IMR (Arkowski et al., 2024). Altogether, these data suggest that CMD may predispose to HFpEF, but particularly when mild diastolic dysfunction is already present. The recently started WISE‐preHFpEF cohort (Takahashi et al., 2025), evaluating the contribution of CMD and myocardial ischemia to the development of HFpEF, is urgently needed to further our mechanistic understanding of the relationship between CMD and the development of HFpEF.

## 3 BIOMARKERS TO DETERMINE PATHOLOGICAL PATHWAYS UNDERLYING CMD AND HFPEF

To gain understanding in the pathogenesis of CMD and HFpEF, several studies investigated circulating biomarkers in CMD (Chakrala et al., 2023; Prescott et al., 2023; Schroder et al., 2019; Suhrs et al., 2020), early HFpEF (Henkens et al., 2022) and HFpEF (Berger et al., 2024; Cohen et al., 2020; Woolley et al., 2021). Biomarkers associated with CMD can be categorized into distinct pathophysiological processes as listed in Table 1.

**TABLE 1:** Pathophysiological processes and the associated biomarkers involved in coronary microvascular disease.

Pathophysiological process	Associated biomarkers
Inflammation	CRP, hs‐CRP, IL‐6, ST2, PGLYRP1, GDF‐15, TNF‐α, CHI3L1, Rho‐kinase (in neutrophils)
Leukocyte recruitment	ICAM‐1, VCAM‐1, SELE, CCL16, CXCL16, CD40‐ligand
Oxidative Stress	Glutathione, PON3, MPO
Coagulation/platelet function	PAI‐1, tPA, D‐dimer, suPAR, serotonin, homocysteine
Endothelial function	ADMA, vWF, Renalase, Endothelin‐1, Adrenomedullin, EPHB4
Blood Pressure/kidney function	Renin, ACE2, Klotho, Uric acid
Cardiac Stress and remodeling	BNP, NT‐proBNP, Cardiac bridge integrator 1, MMP9
Obesity/diabetes/metabolism	LDL, Apolipoprotein C, Omega‐3 fatty acids, SERPINA12, FABP4

Abbreviations: (hs)‐CRP, (high sensitive) C‐reactive protein; ACE2, angiotensin‐converting enzyme 2; ADMA, asymmetric dimethyl arginine; BNP, brain natriuretic peptide; CCL16, CC motif chemokine ligand 1; CHI3L1, chitinase 3‐Like 1; CXCL16, CD40‐ligand, cluster of differentiation 40 ‐ligand; EPHB4, ephrin B4; FABP4, fatty acid‐binding protein 4.; GDF‐15, growth/differentiation factor 15; ICAM‐1, intercellular adhesion molecule 1; IL‐6, interleukin‐6; LDL, Low‐density lipoprotein; MMP9, matrix metalloproteinase 9; MPO, myeloperoxidase; NT‐proBNP, N‐terminal prohormone of brain natriuretic peptide; PAI‐1, plasminogen activator inhibitor type 1; PGLYRP1, peptidoglycan recognition protein 1; PON3, paraoxonase 3; SELE, E‐selectin; SERPINA12, serine protease inhibitor A12; suPAR, soluble urokinase plasminogen activator receptor; TNF‐α, tumor necrosis factor α; tPA, tissue plasminogen activator; VCAM‐1, vascular cell adhesion molecule 1; vWF, von Willebrand Factor.

**FIGURE 1:**
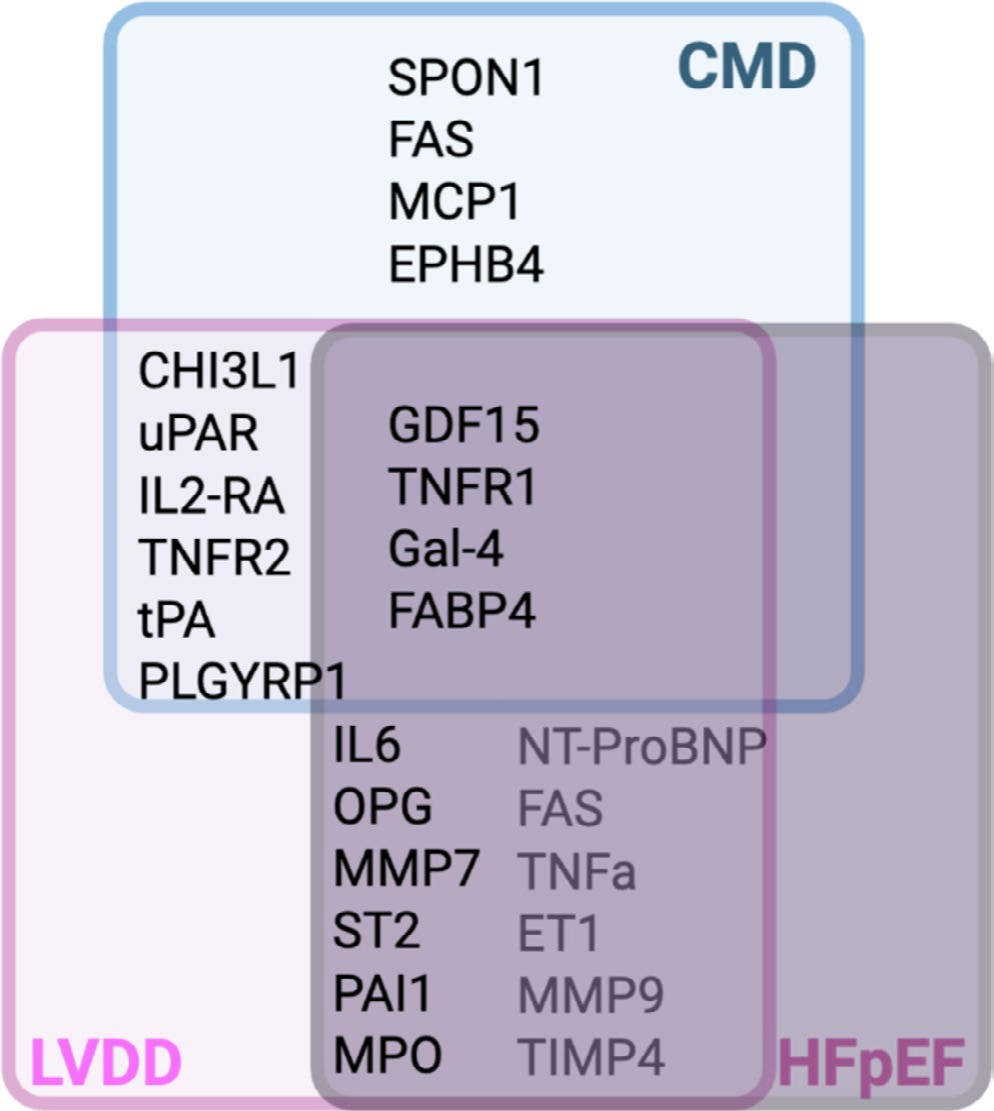
Biomarkers associated with key processes in coronary microvascular disease (CMD) and their overlap with diastolic dysfunction and heart failure with preserved ejection fraction (HFpEF). CHI3L1, chitinase 3‐like 1; CTSD, cathepsin D; CTSZ, cathepsin Z; EPHB4, ephrin B4; ET1, endothelin 1; FABP4, fatty acid‐binding protein 4; FAS, fas cell surface death receptor; Gal‐4, galectin 4; GDF‐15, growth/differentiation factor 15; IL2‐RA, interleukin 2 receptor alpha‐subunit; IL‐6, interleukin‐6; LVDD, left ventricular diastolic dysfunction; MCP1, monocyte chemoattractant protein 1; MMP7, matrix metalloproteinase 7; MMP‐9, matrix metalloproteinase 9; MPO, myeloperoxidase; NT‐proBNP, N‐terminal prohormone of brain natriuretic peptide; OPG, osteoprotegerin; PAI‐1, plasminogen activator inhibitor type 1; PLGYRP1, peptidoglycan recognition protein 1; RARRES2, retinoic acid receptor responder 2; ROS, reactive oxygen species; SPON1, spondin; ST2, suppressor of tumorigenicity 2; suPAR, soluble urokinase plasminogen activator receptor; TIMP4, tissue inhibitor of matrix metalloproteinases 4; TNFR1, tumor necrosis factor receptor 1; TNFR2, tumor necrosis factor receptor 2; TNF‐α, tumor necrosis factor α; tPA, tissue plasminogen activator. Created in BioRender. Merkus, D. (2025) https://BioRender.com/nbpckrh.

After correction for clinical CMD risk factors, a group of eight of 18 biomarkers—CC motif chemokine ligand 16 (CCL16), CXC motif ligand 16 (CXCL16), growth and differentiation marker 15 (GDF15), peptidoglycan recognition protein 1 (PGLYRP1), suppression of tumorigenicity 2 (ST2), soluble urokinase plasminogen activator receptor (suPAR), tumor necrosis factor receptor 1 (TNFR1), and TNF receptor superfamily 10C (TNFRSF10C) remained associated with CFR in women CMD (Schroder et al., 2019). These biomarkers mostly related to TNFα‐IL6‐CRP signaling, suggesting an important role of inflammation in CMD. To further address whether circulating biomarkers could be associated with CMD and/or diastolic dysfunction specifically, the relation between biomarkers and CMD and/or diastolic dysfunction was assessed in a group of women with ANOCA, with and without diabetes and healthy controls (Suhrs et al., 2020). Distinct sets of biomarkers were associated with CMD, diastolic dysfunction, or both (Figure 1) (Suhrs et al., 2020). In another cohort of patients with non‐acute potentially cardiac‐related dyspnea, the HELPFUL study, four phenogroups were identified based on increasing severity of diastolic dysfunction using the HFA‐PEFF criteria (Henkens et al., 2022). Diastolic function was classified as (1) normal cardiac structure and function, (2) diastolic dysfunction with functional LV abnormalities, (3) diastolic dysfunction with functional or structural LV abnormalities, and (4) diastolic dysfunction with functional and structural LV abnormalities in combination with elevated BNP. Biomarker analysis revealed 32 differentially regulated biomarkers, clearly discriminating between these four groups. Of the significant proteins, nine overlapped with biomarkers associated with the biomarkers for CMD and diastolic dysfunction from the study of Suhrs et al. (Suhrs et al., 2020), four with biomarkers of CMD alone, and one with diastolic dysfunction alone (Figure 1). Several of these biomarkers also correlated with prognosis in a HFpEF cohort (Chirinos et al., 2020), underlining the shared pathological mechanisms of CMD and HFpEF. Machine learning analyses of biomarker studies in HFpEF further show patient subclusters (Woolley et al., 2021), with inflammation and activation of innate and adaptive immunity being most differentially regulated among these different subclusters. The highest pathway activation was observed in patients with both diabetes and CKD (Woolley et al., 2021), consistent with studies showing upregulation of the TNFα‐signaling pathway in plasma of HFpEF patients with these comorbidities, as well as with their poor clinical prognosis (Cohen et al., 2020).

Interestingly, predictive biomarkers of CMD in HFpEF patients appear to differ by sex. In men, pathways related to inflammation, chemokine, cytokine and interleukin signaling predominate. In contrast, women show greater activation of pathways associated with coagulation, PI3‐kinase, plasminogen activating cascade and TGF‐β signaling are more prominent (Chandramouli et al., 2022).

## 4 MYOCARDIAL CHANGES IN CMD AND HFPEF

Biomarkers of systemic inflammation, innate and adaptive immunity, endothelial dysfunction, and altered metabolism correlate with the severity of disease and prognosis in both CMD and HFpEF. However, analyses of myocardial and coronary microvascular structure and function are required to understand the pathogenic processes ongoing within the heart. Post‐mortem histology shows reduced capillary density in the myocardium of HFpEF patients that correlated with increased interstitial fibrosis (Mohammed et al., 2015), while endocardial biopsies showed infiltration of inflammatory cells (Hahn et al., 2020) as well as increased expression of pro‐inflammatory adhesion molecules (VCAM1, ICAM1, E‐selectin) on the endothelial cells (Franssen et al., 2016).

Adipose tissue is increasingly recognized as an endocrine organ and source of inflammatory mediators. In metabolic derangement, the quantity and composition of adipose tissue change. The amount of EAT has been shown to be a predictor of both coronary microvascular dysfunction (Abusnina et al., 2025; Huang et al., 2022) and HFpEF (Janssen‐Telders et al., 2025; Menghoum et al., 2025; Nakanishi et al., 2017). An altered balance of adipokines (e.g., more leptin, activin‐A, interleukin 6 (IL6), plasminogen activator inhibitor‐1 (PAI‐1), less adiponectin) in patients with metabolic derangement promotes microvascular endothelial dysfunction (Cheng & Daskalakis, 2015; Janssen‐Telders et al., 2025). Indeed, adiponectin‐knockout mice exhibited coronary microvascular dysfunction and impaired isovolumetric relaxation following exercise training (Caldwell et al., 2021), suggesting that adiponectin has a protective role in both the coronary microvasculature and the heart. In addition, an inflammatory state of VAT or EAT induces pro‐inflammatory macrophage polarization and recruitment into the heart which may cause deterioration of microvascular structures and induce interstitial fibrosis (Schiattarella et al., 2022), thereby contributing to the development of HFpEF.

In order to intervene with disease pathogenesis, pathophysiological pathways need to be identified in an earlier state of the disease. Animal models of CMD (Sorop et al., 2020) and HFpEF (van Ham et al., 2022) form a critical step in understanding pathogenesis and testing potential novel therapies. However, it is imperative that these animal models present with critical features observed in the disease in patients (Gomberg‐Maitland et al., 2016). For this review, we will focus on models in which both CMD and diastolic function have been reported. Inflammation, oxidative stress, and endothelial dysfunction due to exposure to risk factors like diabetes, obesity, aging, and chronic kidney disease play a central role in the development of CMD and HFpEF (Woolley et al., 2021). Oxidative stress reduces the bioavailability of NO, both directly and indirectly through uncoupling of endothelial nitric oxide synthase (eNOS). The subsequent reduction in soluble guanylyl cyclase (sGC) activation in the cardiomyocytes results in hypophosphorylation of titin, stiffening of the cardiomyocytes, and impaired myocardial relaxation (Paulus & Tschope, 2013). Loss of NO may also directly contribute to impaired myocardial perfusion, particularly under stress such as exercise. These processes already play a role in early disease as evidenced by preclinical studies evaluating the effect of exposure to these risk factors on inflammation, cardiac function, and perfusion. Thus, pigs with metabolic derangement and CKD showed elevated levels of TNF‐α and IL‐6, evidence for diastolic dysfunction (Chade & Eirin, 2022; Sorop et al., 2018) as well as reduced CFR and impaired myocardial perfusion (van de Wouw et al., 2020) that was mediated by loss of NO‐dependent vasodilation at rest and during exercise and accompanied by reduced eNOS expression, eNOS uncoupling, and impaired endothelium‐dependent vasodilation of isolated coronary small arteries (van de Wouw et al., 2021). Similar to swine with CKD alone (Sen et al., 2024), the reduction in CFR in these animals with multiple comorbidities was due to an increase in basal CBF (van de Wouw et al., 2021), whereas maximal CBF was not different, indicating functional rather than structural CMD. Histological analyses of the myocardium showed an increase in oxidative stress both in the cardiomyocytes and in the coronary microvasculature (Sen et al., 2024), which was accompanied by a reduction in total antioxidant capacity (Sen et al., 2024; van Drie et al., 2024). Intriguingly, exposing cultured myocardial slices of these animals to H_2_O_2_ impaired contractile function (Sen et al., 2024), while scavenging of reactive oxygen species (ROS) in vivo reduced CBF, suggesting that ROS served as vasodilators and may have contributed to the higher basal CBF (van Drie et al., 2024). Molecular analyses of the myocardium of swine with metabolic derangement and CKD suggested impaired VEGF signaling, which was accompanied by reduced subendocardial capillary density (Eirin & Chade, 2023). Parallel swine studies provided evidence for alterations in signaling pathways involved in extracellular matrix (ECM) remodeling and showed interstitial fibrosis (Chade et al., 2024; Chade & Eirin, 2022; Sen et al., 2024).

Mice on a western diet exhibited echographic evidence for diastolic dysfunction and hypertrophy. CMD, that is, impaired NO‐mediated vasodilation to ACh, increased vasoconstriction to thromboxane, and oxidative stress, was accompanied by upregulation of VCAM in the coronary vasculature as well as an increased stiffness of the endothelial cells as measured with atomic force microscopy (Dona et al., 2023). RNA sequencing showed upregulation of pathways associated with inflammation and leukocyte migration. scRNA sequencing of non‐myocyte cardiac cell populations revealed induction of inflammatory pathways in endothelial cells while resident myocardial inflammatory cells showed an increase in pathways associated with antigen processing and presentation as well as leukocyte activation. Interestingly, CMD, diastolic dysfunction, and molecular changes in the myocardium could be reversed by mineralocorticoid receptor (MR) inhibition as well as by smooth muscle‐specific MR knockout (Dona et al., 2023). However, contrary to observations in swine and humans, there was no evidence of interstitial fibrosis, neither at the molecular level nor histologically.

## 5 MITOCHONDRIAL CHANGES AND MECHANOENERGETIC DECOUPLING IN HFPEF

The heart is the organ with the highest metabolic rate in our body. ATP generated by the cardiac mitochondria is used for cardiac contraction (60%–70%) and the rest is mostly used for ion pumps required to maintain membrane potential. Less than 5% of ATP is derived from anaerobic glycolysis, and hence, most of the ATP (>95%) is generated by oxidative phosphorylation in the mitochondria. The heart is omnivorous and metabolically flexible, meaning that cardiomyocytes can utilize different substrates, including carbohydrates (20%–40%), fatty acids (40%–60%), ketone bodies (10%–15%), and amino acids (<2%) to generate ATP (Sun et al., 2024).

In HFpEF, myocardial energetics undergo several maladaptive changes. Increased afterload raises ATP demand, while mechanical efficiency decreases. As a result, more oxygen, and hence higher coronary blood flow, are required for the same amount of work; yet coronary microvascular dysfunction simultaneously impairs the coupling of myocardial blood flow to myocardial work (AbouEzzeddine et al., 2019; van de Wouw et al., 2020). Lower phosphocreatine to ATP ratios further suggest an energy deficit (Aksentijevic et al., 2025; Mericskay et al., 2025).

ATP and cytosolic Ca^2+^ cycling are essential for regulation of cardiac contraction and relaxation. However, oxidative phosphorylation in the mitochondria is also regulated both by Ca^2+^ and ADP, enabling the close coupling between energy production and cardiac mechanics referred to as mechano‐energetic coupling (Aksentijevic et al., 2025; Maack, 2023). Impaired mitochondrial Ca^2+^ uptake may result in compromised ATP generation and thereby cause a breakdown in the effective transfer of metabolic energy (from oxidative phosphorylation) into mechanical effort (e.g., muscular contraction), so‐called mechanoenergetic decoupling (Aksentijevic et al., 2025; Maack, 2023). In HFpEF, defects in mitochondrial Ca^2+^ uptake have been associated with alterations in the mitochondrial Ca^2+^ uniporter (MCU) (Alves‐Figueiredo et al., 2024). For example, in a mouse model of type 1 diabetes, reduced MCU expression and mitochondrial Ca^2+^ uptake were ameliorated by AAV‐mediated MCU upregulation, which restored mitochondrial Ca^2+^ uptake, pyruvate dehydrogenase activity, mitochondrial respiration, and both systolic and diastolic cardiac function (Suarez et al., 2018). Conversely, increased mitochondrial Ca^2+^ levels and uptake were also reported in a ZFS1 obese rat model of metabolic HFpEF, with no change in MCU (Miranda‐Silva et al., 2020). Hence, further investigation is needed to clarify whether alterations in mitochondrial Ca^2+^ handling contribute to mechano‐energetic uncoupling and the progression of HFpEF.

Metabolic dysregulation due to obesity and insulin resistance results in an oversupply of fatty acids and subsequent lipid overload in the myocardium (Mericskay et al., 2025), thereby inducing a shift in metabolic substrate and reducing metabolic flexibility (Shehadeh et al., 2025). Oxidative damage caused by increased fatty acid oxidation (FAO) in mitochondrial membranes increases proton leakage. Such proton “leak” across the inner mitochondrial membrane results in the bypassing of ATP synthase, the enzyme responsible for the creation of ATP. This results in a decrease in the efficiency of ATP generation and an increase in oxygen demand without equivalent ATP synthesis, linking cardiometabolic diseases to energy deficits. Simultaneously, lipid overload, together with the impaired mitochondrial bioenergetics and a reduction in the mitochondrial quality control mechanism, results in excessive production of ROS at respiratory chain complexes I and III, while simultaneously lowering antioxidant capacity by impaired production of NADH and NADPH, further exacerbating oxidative stress (Cui et al., 2025; Mericskay et al., 2025).

Studies in swine with chronic kidney disease (CKD) demonstrate that hypertrophied myocardium exhibits reduced mitochondrial matrix density, mitochondrial uncoupling (evidenced by increased H_2_O_2_ production), and diminished ATP synthesis (Nargesi et al., 2021). Proteomic analyses of porcine myocardium reveal downregulation of mitochondrial structural and functional proteins in swine with CKD‐induced CMD and diastolic dysfunction (Sen et al., 2024), while RNAseq data suggest downregulation of genes related to mitochondrial proteins (Chade et al., 2024; Chade & Eirin, 2022). Also in the coronary microvasculature, mitochondrial dysfunction induces oxidative stress and contributes to CMD (Gutierrez‐Huerta et al., 2025; Yuan et al., 2018). Thus, mitochondrial dysfunction is a shared mechanism linking both CMD and HFpEF.

There is emerging evidence that targeting mitochondrial dysfunction through restoration of cardiolipin, an important constituent of mitochondria, can improve microvascular (Yuan et al., 2018) as well as cardiac dysfunction (Eirin et al., 2016) induced by CKD, suggesting that targeting mitochondrial dysfunction can be a valuable strategy to treat CMD and HFpEF. Furthermore, inducing a metabolic shift by administration of ketones (beta‐hydroxybutyrate, BHB) was recently shown to improve mitochondrial function as well as cardiac function in a rodent HFpEF model (Deng et al., 2021; Liao et al., 2021).

A recent clinical study evaluated the effect of 2 weeks of oral BHB administration in HFpEF patients and showed improved LV end‐diastolic pressure volume relations, indicative of improved diastolic compliance that was accompanied by reduced capillary wedge pressure at rest and during exercise and an increase in cardiac output at rest (Gopalasingam et al., 2024). Notably, SGLT‐2 inhibitors, which are known to induce mild ketosis, may exert some of their beneficial effects through similar metabolic mechanisms.

## 6 EFFECT OF HFPEF TREATMENT ON CMD

Drugs that interfere with the renin–angiotensin system, including angiotensin receptor blockers (ARBs), angiotensin‐converting enzyme (ACE) inhibitors, combinations of ARBs with neprilysin inhibitors (ARNIs) as well as mineralocorticoid receptor antagonists (MRAs) have been used to treat HFpEF. Large clinical trials such as the PARAGON‐HF and the PARAGLIDE‐HF trials have demonstrated that treatment with ARNIs shows greater reduction in NT‐proBNP and high‐sensitive troponin T (HsTNT) with reduced rehospitalization for heart failure as compared to ARB or ACE‐inhibitor alone (Cunningham et al., 2020; Gori et al., 2021; Vaduganathan et al., 2023). Also, in patients with CMD, interfering with the renin–angiotensin system has been shown to improve CFR (Abouzid et al., 2024; Higuchi et al., 2007). However, to the best of our knowledge, there is no direct evidence demonstrating improvements in coronary microvascular function in HFpEF patients treated specifically with drugs targeting the renin–angiotensin system.

Sodium glucose transporter 2 inhibitors (SGLT2 inhibitors) have become the cornerstone of HFpEF treatment. These drugs were originally developed to promote glycosuria in diabetes, but have since shown benefits on multiple processes contributing to HFpEF. Thus, SGLT2 inhibitors promote natriuresis and reduce glomerular damage, reduce blood pressure and metabolic demand, induce ketosis and weight loss, and alleviate inflammation and oxidative stress (Chen et al., 2025). In the heart, they reduce myofilament stiffness and myocardial fibrosis. Several large clinical trials (EMPEROR‐PRESERVED, DELIVER, SCORED) have shown that SGLT2 inhibitors improve cardiac function and reduce morbidity and mortality in HFpEF (Chen et al., 2025; Jaiswal et al., 2023). However, the effect of SGLT2 inhibition on the coronary microvasculature is less well established. Preclinical studies show a vasodilator effect on the coronary microcirculation (Chen et al., 2025) which is associated with an increase in NO production (Capece et al., 2024). Interestingly, a few studies investigate the effect of SGLT2 inhibition on CMD in HFpEF patients. Most studies show an improvement of CFR, due to a decrease in basal CBF (Chen et al., 2025; Lauritsen et al., 2021; Leccisotti et al., 2022). However, this appears specific to HFpEF, as it was not observed in diabetic patients (Jurgens et al., 2021).

Glucagon‐like‐peptide 1 (GLP1) is a hormone that regulates blood glucose levels by modulating insulin sensitivity but also has beneficial effects on the cardiovascular system beyond control of glucose level. Preclinical data show that GLP‐1 agonists improve endothelial function by increasing NO production and reducing endothelin‐1, as well as by reducing oxidative stress and attenuating expression of inflammation‐induced expression of adhesion molecules on the endothelium. Thus, GLP‐1 agonists act mainly on the inflammatory response of the endothelial cells, and the combination of GLP‐1 with insulin has also been associated in human patients with a significant reduction in systemic markers of vascular inflammation, including soluble intercellular adhesion molecule (sICAM‐1), plasma 8‐iso‐prostaglandin F2α (8‐iso‐PGF2α), nitrotyrosine, and IL‐6 (Karakasis et al., 2025). Contrary to SGLT2 inhibitors, GLP1 agonists seem to increase rather than decrease basal blood flow, which is accompanied by an increase in high‐energy phosphates in the myocardium (Chowdhary et al., 2024). Similarly, maximal blood flow was increased in response to GLP‐1 agonism in diabetic patients without a history of CVD (Chowdhary et al., 2024). How GLP‐1 agonists impact the development/progression of CMD in HFpEF remains to be established.

## 7 CONCLUSION AND FUTURE PERSPECTIVES

**FIGURE 2:**
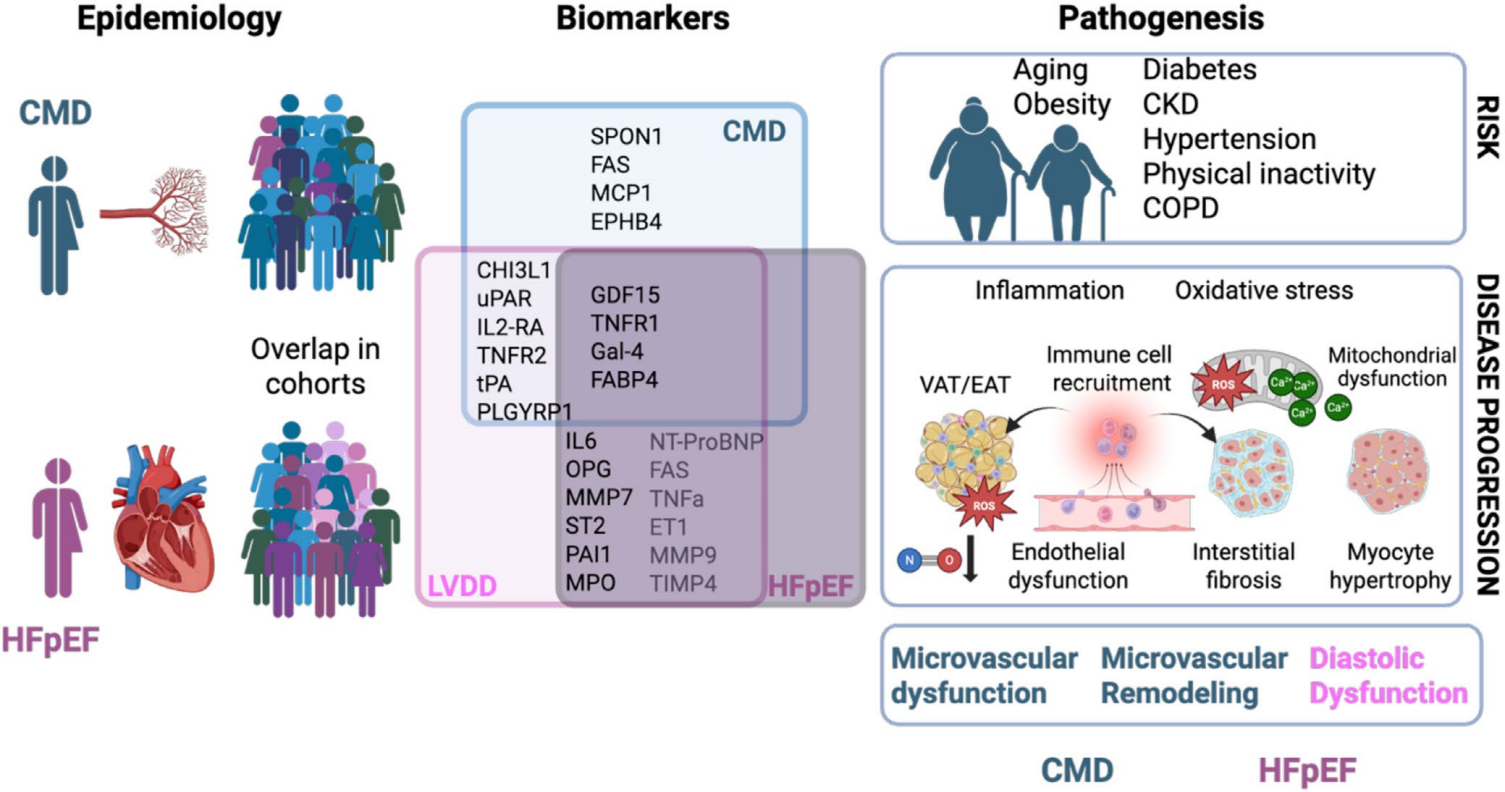
Coronary microvascular disease (CMD) and heart failure with preserved ejection fraction show strong overlap in patient cohorts, due to shared risk factors, resulting in inflammation, oxidative stress, mitochondrial dysfunction, and endothelial dysfunction, leading to increased interstitial fibrosis and myocyte hypertrophy. The shared pathogenesis is also evidenced by shared biomarkers. CHI3L1, chitinase 3‐like 1; CKD, chronic kidney disease; COPD, chronic pulmonary obstructive disease; CTSD, cathepsin D; CTSZ, cathepsin Z; EAT, epicardial adipose tissue; EPHB4, ephrin B4; ET1, endothelin 1; FABP4, fatty acid binding protein 4; FAS, Fas cell surface death receptor; Gal‐4, galectin 4; GDF‐15, growth/differentiation factor 15; IL2‐RA, interleukin 2 receptor alpha‐subunit; IL‐6, interleukin‐6; LVDD, left ventricular diastolic dysfunction; MCP1, monocyte chemoattractant protein 1; MMP7, matrix metalloproteinase 7; MMP‐9, matrix metalloproteinase 9; MPO, myeloperoxidase; NT‐proBNP, N‐terminal prohormone of brain natriuretic peptide; OPG, osteoprotegerin; PAI‐1, plasminogen activator inhibitor type 1; PLGYRP1, peptidoglycan recognition protein 1; RARRES2, retinoic acid receptor responder 2; ROS, reactive oxygen species; SPON1, spondin; ST2, suppressor of tumorigenicity 2; suPAR, soluble urokinase plasminogen activator receptor; TIMP4, tissue inhibitor of matrix metalloproteinases 4; TNFR1, tumor necrosis factor receptor 1; TNFR2, tumor necrosis factor receptor 2; TNF‐α, tumor necrosis factor α; tPA, tissue plasminogen activator; VAT, visceral adipose tissue. Created in BioRender. Merkus, D. (2025) https://BioRender.com/nbpckrh.

The prevalence of diabetes, obesity, and chronic kidney disease is increasing in the general population, which together with an increasingly sedentary lifestyle has contributed to the growing burden of both CMD and HFpEF. Epidemiological studies show a very high prevalence of CMD in HFpEF populations, while fewer studies have explored CMD as a causality to HFpEF. Biomarker profiles of CMD and HFpEF show a large overlap, suggesting shared pathomechanisms in both diseases. Metabolic dysregulation, inflammation, and oxidative stress disrupt the endocrine function of adipose tissue and contribute to endothelial dysfunction, thereby playing a central role in the development of HFpEF. These fat depots play a critical role in linking metabolic disturbances to coronary microvascular and myocardial abnormalities observed in HFpEF. Additionally, mitochondrial dysfunction characterized by reduced substrate flexibility, impaired energy production, and disrupted Ca^2+^ homeostasis contributes to increased reactive oxygen species (ROS) production and impaired antioxidant defenses (Figure 2).

Interventions targeting metabolic derangement, particularly SGLT2 inhibitors and GLP1 agonists, have become the cornerstone of HFpEF treatment. Although their impact on inflammation and cardiac function has been well established, their impact on mitochondrial function, Ca‐homeostasis, and coronary microvascular function is less well known and should be the subject of further studies.

## FUNDING INFORMATION

This work was supported by German Center for Cardiovascular Research (DZHK81Z0600207 to D.M. and P.S.) as well as the Dutch CardioVascular Alliance: An initiative with support of the Dutch Heart Foundation (2020B008 RECONNEXT for D.M.).

## CONFLICT OF INTEREST STATEMENT

None of the authors has a conflict of interest to declare.

## DISCLOSURE STATEMENT

Daphne Merkus is an Associate Editor at Physiological Reports and was blinded from reviewing or making decisions for the manuscript. Another Editor oversaw the manuscript process for this article.

## Data Availability

The manuscript is a review and contains no original data.
